# Multimorbidity in Hospitalized Patients Admitted to General Practice Departments and Its Implications for the General Practice Healthcare System: A Four-Year Longitudinal Study in China

**DOI:** 10.3389/fpubh.2021.760792

**Published:** 2021-12-20

**Authors:** Zhihan Zhou, Meng Shi, Mengzhu Liu, Jianqin Gu, Clifford Silver Tarimo, Jian Wu, Yudong Miao

**Affiliations:** ^1^Department of Political Communication, Guangming School of Journalism and Communication, China University of Political Science and Law, Beijing, China; ^2^Department of Health Management, School of Medicine and Health Management, Tongji Medical College, Huazhong University of Science and Technology, Wuhan, China; ^3^Intelligent Hospital Engineering Research Center of Henan Provincial People's Hospital, People's Hospital of Zhengzhou University, Zhengzhou, China; ^4^Healthy Life-Style Research Center, School of Medicine, Southern University of Science and Technology, Shenzhen, China; ^5^College of Public Health, Zhengzhou University, Zhengzhou, China; ^6^Center of Health Development, College of Public Health, Zhengzhou University, Zhengzhou, China

**Keywords:** multimorbidity, the general practice department, hospitalized patients, China, general practice healthcare system

## Abstract

**Objective:** China and many developing countries has placed high expectations on the general practice healthcare system in terms of lowering medical costs and improving the health status of the multimorbid population in recent years. However, the prevalence of multimorbidity among inpatients attending the general practice department of hospitals and its policy implications are largely unknown. The current study aimed to analyze the prevalence of comorbidities among inpatients attending the general practice department of the tertiary Grade-A Hospitals in China, and put forward evidence-based policy recommendations.

**Methods:** Between December 2016 and November 2020, 351 registered general practitioners from 27 tertiary hospitals were selected, and their direct admissions were evaluated. The rate and composition ratio were used for descriptive analysis of the clinical and epidemiological characteristics of multimorbidity. A backward stepwise algorithm was used to explore independent variables. The absence of multicollinearity and plausible interactions among variables were tested to ensure the robustness of the logistic regression model. The pyramid diagram was used to show the link between gender and the involved human body system in multimorbidity.

**Results:** Multimorbidity was present in 93.1% of the 64, 395 patients who were admitted directly. Multimorbidity was significantly more prevalent in patients aged 45–59 years (OR=3.018, 95% CI=1.945–4.683), 60–74 years (OR = 4.349, 95% CI = 2.574–7.349), ≥75 years (OR = 7.804, 95% CI = 3.665–16.616), and those with body mass index (BMI) ≥ 28 kg/m^2^ (OR = 3.770, 95% CI = 1.453–9.785). The circulatory system was found to be the most commonly involved human body system in multimorbidity, accounting for 79.2% (95% CI = 78.8–79.5%) of all cases. Significant gender inequity was further observed in the involved human body system in multimorbidity.

**Conclusion:** Multimorbidity is likely common among the inpatients attending the general practice department of hospitals in China and many developing countries, with significant gender inequity in the involved human body systems. Effective countermeasures include establishing a GP-PCIC multimorbidity prevention and control model and enhancing the multimorbidity of elderly and obese patients at both the clinical and healthy lifestyle levels. The diagnosis and treatment capabilities of GPs on the circulatory, endocrine, metabolic, digestive, and respiratory systems should be prioritized.

## Introduction

Preventing and controlling non-communicable chronic diseases is a critical global health issue that requires immediate attention. As the largest developing country in transition, chronic diseases have already emerged as China's most challenging health threat (1, 2). According to the World Bank, chronic diseases account for more than 80% of the 10.3 million deaths caused by various factors each year in China and account for 68.6% of the disease burden. By the year 2030, China's population over the age of 40 is expected to grow by 2–3 times in terms of chronic diseases (cardiovascular diseases, chronic obstructive pulmonary disease, diabetes, and lung cancer) (3). The situation is likely to get worse as the population ages. China's elderly population will grow from 115 million today to approximately 240 million in 2030, while the super-aged population (over 80 years old) is expected to grow from 12 million in 2000 to over 40 million (4).

Numerous studies conducted in western countries indicate that a large proportion of people present a multimorbid condition (5), which refers to patients presenting two or more chronic diseases concurrently (6, 7). As multimorbidity progresses, the interaction of chronic diseases wreaks greater havoc (8), necessitating more comprehensive and continuous clinical treatment (9–11). However, the majority of guidelines are developed and implemented using a single disease approach, which treats diseases in isolation (12). Patients with multiple chronic diseases are frequently treated by a variety of different healthcare specialists (for each chronic disease), particularly in China, where specialist care is widespread (13).

China has placed high expectations on the general practice healthcare system since the 2009 healthcare reform in terms of lowering medical costs and improving the health status of the multimorbid population. China now has over 3,65,000 GPs, including 2,10,000 registered GPs and 1,55,000 qualified GPs (14). In recent years, the number of general practitioners has grown much faster than the number of specialists, but this has been accompanied by a rapid decline in qualification rates [(15–17), shown in Supplementary Figure 1]. To facilitate the development of an effective and efficient general practice healthcare system based on qualified general practitioners, the government began requiring all tertiary hospitals to establish general practice departments in 2014 and designating them as engines for increasing general medical service capacity and quality. This means that strengthening the service capacity and quality of GPs in tertiary hospitals will contribute to the overall improvement of China's general practice service system.

Typically, the epidemiology of multimorbidity among the inpatients admitted to the general practice departments of tertiary hospitals are more complex, providing a unique perspective on inpatient multimorbidity and a precise approach to promoting the general practice healthcare system. Although a few studies have been conducted among Chinese older adults, the epidemiology of multimorbidity among inpatients admitted to the general practice departments of tertiary hospitals still remains unknown (18, 19). Therefore, the purpose of this study was to examine the clinical epidemiological characteristics of 64,395 hospitalized patients in the general practice departments of 27 tertiary grade-A hospitals in China (shown in Supplementary Table 1), and to make evidence-based policy recommendations for effectively promoting multimorbidity prevention and control, as well as GPs' diagnosis and treatment capabilities.

## Methodology

### Study Time and Settings

The study started in December 2016 and accomplished in November 2020. We followed up the general practice departments of 27 tertiary grade-A hospitals in China that had the earliest standardized training bases for general practice residents.

### Participant Recruitment

A two-stage cluster sampling method was used to obtain GPs and their hospitalized patients. At the first stage, all GPs who were (1) qualified and registered as GP; (2) doctors-in-charge or above; and (3) willing to participate and sign the informed consent form were included. At the second stage, all inpatients of each GP were followed, but patients transferred from other departments, those with repeated hospitalizations, those referred from other hospitals, and those who refused to participate were excluded.

### Data Collection

We firstly urged the GPs to collect electronic medical record information through the hospital information system. We contacted GPs and patients via telephone to confirm any ambiguous or missing data. This study collected information of the participants on gender, age, marital status, education level, health insurance, occupation, past history, BMI, smoking, alcohol consumption, admission diagnosis and discharge diagnosis according to the International Classification of Diseases (ICD-10) standards, disease counts, and other clinical and epidemiological characteristics as well as influencing factors for multimorbid condition. Based on the recommended definition in international multimorbidity studies (5, 20), multimorbidity in the current study was defined and measured as the simultaneous occurrence of several chronic conditions in the same patient (21).

### Patient and Public Involvement

Patients and/or the public were not involved in the design, or conduct, or reporting or dissemination plans of this research.

### Statistical Analysis

The proportions of morbidities across groups were compared using Chi-squared test. The rate and composition ratio were used for descriptive analysis of the clinical and epidemiological characteristics of multimorbidity. A backward stepwise algorithm was used to explore independent variables. The absence of multicollinearity and plausible interactions among variables were tested to ensure the robustness of the logistic regression model. The pyramid diagram was used to show the link between gender and the involved human body system in multimorbidity. Differences were regarded as statistically significant if *P* values were < 0.05. Statistical analyses were performed in IBM SPSS Statistics 20.0 (Chicago, IL, USA).

## Results

### Prevalence of Multimorbidity and the Distribution Across Groups

Three hundred and fifty one GPs and their 64, 395 hospitalized patients were included in the analysis. The age range of study participants were 5 to 98 years, with an average age of 60 years. 4,455 cases (6.9%) had one chronic disease, and 59, 940 (93.08%) had two or more chronic diseases. The prevalence of multimorbidity in patients over 75 years was 97.5%. The prevalence of multimorbidity among participants with normal body mass index (BMI) (18.5 ≤ BMI <23.9, 89.4%) was significantly lower than those with abnormal BMI (BMI <18.5, 90.1%; 24 < BMI BMI ≤ 27.9, 94.4%; BMI ≤ 28, 97.2%; *P* < 0.01). The prevalence of multimorbidity in the fully out-of-pocket group was lower than in basic health insurance and other groups (Fully out-of-pocket, 88.4%; MIUW, 95.0%; MIUR, 92.3%; NRCMI, 91.0%; CMI, 95.7%; *P* < 0.01). The prevalence of multimorbidity among retired personnel was the highest, accounting for 97.6% (civil servants, 95.9%; corporate personnel, 88.9%; farmers, 93.5%; self-employed workers, 91.8%; others, 92.2%; *P* < 0.01). The prevalence of multimorbidity was not statistically different among the groups by gender (*P* = 0.548), education level (*P* = 0.619), smoking (P = 0.695) and drinking (*P* = 0.066) (see Table 1).

**Table 1:** Socio-demographic, BMI, lifestyle and multimorbidity characteristics of all study participants.

**Covariates**	**Total**	**Multimorbidity**		***χ^2^***	***P value***
		**Number**	**Percentage(95%*CI)***		
All participants	64,395	59,940	93.1(92.1–94.1)		
**Gender**				0.361	0.548
Male	37,989	35,262	92.8(91.5–94.2)		
Female	26,406	24,678	93.5(91.9–95.0)		
**Age (year)**				111.895	<0.01
<45	9,504	7614	80.1(75.9–84.1)		
45-	21,924	20,601	94.0(92.3–95.6)		
60-	19,791	18,873	95.4(93.8–96.9)		
75-	13,176	12,852	97.5(96.2–98.9)		
**Marital status**				45.152	<0.01
Unmarried	1,134	648	57.1(41.5–72.8)		
Married	58,536	54,891	93.8(92.8–94.8)		
Divorced/Widowed/Unknown	4,725	4,401	93.1(89.4–96.8)		
**Education level**				1.784	0.619
Below elementary school	14,553	13,500	92.8(90.6–95.0)		
junior high school	13,662	12,690	92.9(90.6–95.1)		
High school technical secondary school	13,797	12,717	92.2(89.9–94.5)		
College degree and above	22,383	21,033	94.0(92.3–95.6)		
**Health insurance**				19.407	<0.01
MIUW	20,574	19,548	95.0(93.5–96.6)		
MIUR	9,477	8,748	92.3(89.5–95.1)		
NRCMI	18,387	16,740	91.0(88.9–93.2)		
CMI	11,286	10,773	95.7(93.7–97.7)		
None	4,671	4,131	88.4(83.6–93.3)		
**Occupation**				61.518	<0.01
Civil servant	5,319	5,103	95.9(93.2–98.7)		
Corporate personnel	11,178	9,936	88.9(85.8–91.9)		
Farmer	18,630	17,415	93.5(91.5–03.5)		
Student	432	189	43.8(16.4–71.1)		
Self-employed persons	4,590	4,212	91.8(87.6–95.9)		
Retired personnel	13,473	13,149	97.6(96.2–98.9)		
Others	10,773	9,936	92.2(89.6–94.9)		
**BMI(kg/m** ^**2**^ **)**				33.366	<0.01
<18.5	2,457	2,214	90.1(83.9–96.4)		
18.5-	21,924	19,602	89.4(87.3–91.5)		
24-	27,378	25,839	94.4(93.0–95.8)		
28-	12,636	12,285	97.2(95.7–98.7)		
**Smoking**				0.154	0.695
Smoker/Ever-smoker	25,515	23,814	93.3(91.7–94.9)		
Non-smoker	38,880	36,126	92.9(91.6–94.2)		
**Alcohol consumption**				3.369	0.066
Regular drinker	26,217	24,705	94.2(92.8–95.7)		
Seldom drinker	38,178	35,235	92.3(90.9–93.7)		

*MIUW, medical insurance for urban workers; MIUR, medical insurance for urban residents; NRCMI, the new rural cooperative medical insurance; CMI, commercial medical insurance*.

### Associations Between Multimorbidity and Socio-Demographic, and BMI

The findings indicated that age and obesity were independent risk factors for multimorbidity among the inpatients. The prevalence of multimorbidity increased substantially with age and BMI. Compared with patients in the 45–59-year-old age group, patients in the ≥75-year-old group are 7.804 times more likely to develop multimorbidity (OR = 7.8046, 95%CI = 3.665–16.616). Patients with a BMI of 28 kg/m^2^ were 3.770 times more likely to develop multimorbid condition than patients with a BMI of 18.5 kg/m^2^ (OR = 3.770, 95% CI = 1.453-9.785) (see Table 2).

**Table 2:** Associations between multimorbidity and socio-demographic and BMI.

**Covariates**	**Unadjusted OR**	**95%*CI***	P value^*^	Adjusted OR^**^	**95%*CI***	P value^*^
**Age (year)**
<45	1.000 (ref)		<0.001	1.000 (ref)		<0.001
45-	3.865	2.618–5.708	<0.001	3.018	1.945–4.683	<0.001
60-	5.103	3.312–7.864	<0.001	4.349	2.574–7.349	<0.001
75-	9.846	5.245–18.485	<0.001	7.804	3.665–16.616	<0.001
**Marital status**
Unmarried	1.000 (ref)		<0.001	1.000 (ref)		0.021
Married	11.294	5.983–21.323	<0.001	2.551	1.006–6.467	0.049
Divorced/Unknown	10.187	4.368–23.760	<0.001	1.279	0.413-3.967	0.670
**Health insurance**
MIUW	1.000 (ref)		<0.001	1.000 (ref)		0.190
MIUR	0.630	0.378–1.049	0.076	0.707	0.396–1.264	0.243
NRCMI	0.533	0.351–0.811	0.003	0.623	0.354–1.096	0.100
CMI	1.102	0.627–1.938	0.735	1.093	0.603–1.982	1.093
None	0.402	0.22–0.709	0.002	0.538	0.283–1.025	0.538
**Occupation**
Civil servant	1.000 (ref)		<0.001	1.000 (ref)		0.119
Manager	0.339	0.157–0.732	0.06	0.407	0.184–0.901	0.027
Farmer	0.607	0.281–1.309	0.203	0.650	0.269–1.572	0.339
Student	0.033	0.01–0.111	<0.001	0.355	0.077–1.572	0.186
Self-employed	0.472	0.193–1.153	0.100	0.777	0.298–2.527	0.606
Retired	1.718	0.691–4.269	0.244	0.946	0.354–2.527	0.912
Others	0.502	0.227–1.115	0.90	0.619	0.264–1.451	0.270
**BMI (kg/m** ^2^ **)**
<18.5	1.000 (ref)		<0.001	1.000 (ref)		<0.001
18.5-	0.927	0.449–1.910	0.836	0.740	0.332–1.649	0.461
24-	1.843	0.881–3.856	0.105	1.406	0.622–3.179	0.413
28-	3.841	1.590–9.278	0.003	3.770	1.453–9.785	0.006

* *P values are based on joint tests, which test the overall differences between the individual categories of the corresponding variable*.

** *adjusted for all other covariates (independent variables) listed in the table*.

*CI, confidence interval; OR, odds ratio; Ref, reference. Dependent variable: presence of multimorbidity (1 = Yes; 0 = No)*.

### Clinical Epidemiological Characteristics of Multimorbidity Across Groups by Gender, Age, and BMI

Table 3 showed that only 4,455 patients (6.9%) had only one chronic illness, while 8.8% had two, 13.5% had three, 15.9% had four, and 14.5% had five and 40.4% were diagnosed with six, respectively. The prevalence of chronic diseases among participants with BMI ≥ 24 kg/m^2^ was significantly higher than that of those with normal BMI (18.5 ≤ BMI <23.9, 89.4%). The number of chronic diseases was found to increase with age, particularly in patients with more than six (6) chronic diseases. The proportion of patients with more than six (6) chronic diseases was the highest in the age groups 45–59 years, 60–74 years, and ≥75 years (34.6, 45.4, 59.4% respectively).

**Table 3:** Clinical epidemiological characteristics of multimorbidity across groups with different gender, age and BMI.

**Covariates**	**1**		**2**		**3**		**4**		**5**		**≥6**	
	***n***	**%**	***n***	**%**	***n***	**%**	***n***	**%**	***n***	**%**	***n***	**%**
All participants	4,455	6.9	5,643	8.8	8,694	13.5	10,233	15.9	9,342	14.5	26,028	40.4
**Gender**
Male	2,727	7.2	3,240	8.5	4,806	12.7	5,724	15.1	6,237	16.4	15,255	40.2
Female	1,728	6.5	2,403	9.1	3,888	14.7	4,509	17.1	3,105	11.8	10,773	40.8
**Age (year)**
<45	1,890	19.9	1,485	15.6	1,620	17.0	1,890	19.9	999	10.5	1,620	17.0
45-	1,323	6.0	2,295	10.5	3,429	15.6	3,753	17.1	3,537	16.1	7,587	34.6
60-	918	4.6	1,431	7.2	2,484	12.6	3,186	16.1	2,781	14.1	8,991	45.4
75-	324	2.5	432	3.3	1,161	8.8	1,404	10.7	2,025	15.4	7,830	59.4
**BMI(kg/m** ^**2**^ **)**
<18.5	243	9.9	243	9.9	486	19.8	378	15.4	243	9.9	864	35.2
18.5-	2,322	10.6	2,430	11.1	3,159	14.4	3,645	16.6	2,970	13.5	7,398	33.7
24-	1,539	5.6	2,133	7.8	3,456	12.6	4,347	15.9	4,158	15.2	11,745	42.9
28-	351	2.8	837	6.6	1,593	12.6	1,863	14.7	1,971	15.6	6,021	47.6

### Clinical Characteristics of Multimorbidity Involved in the Body System

**Figure 1:**
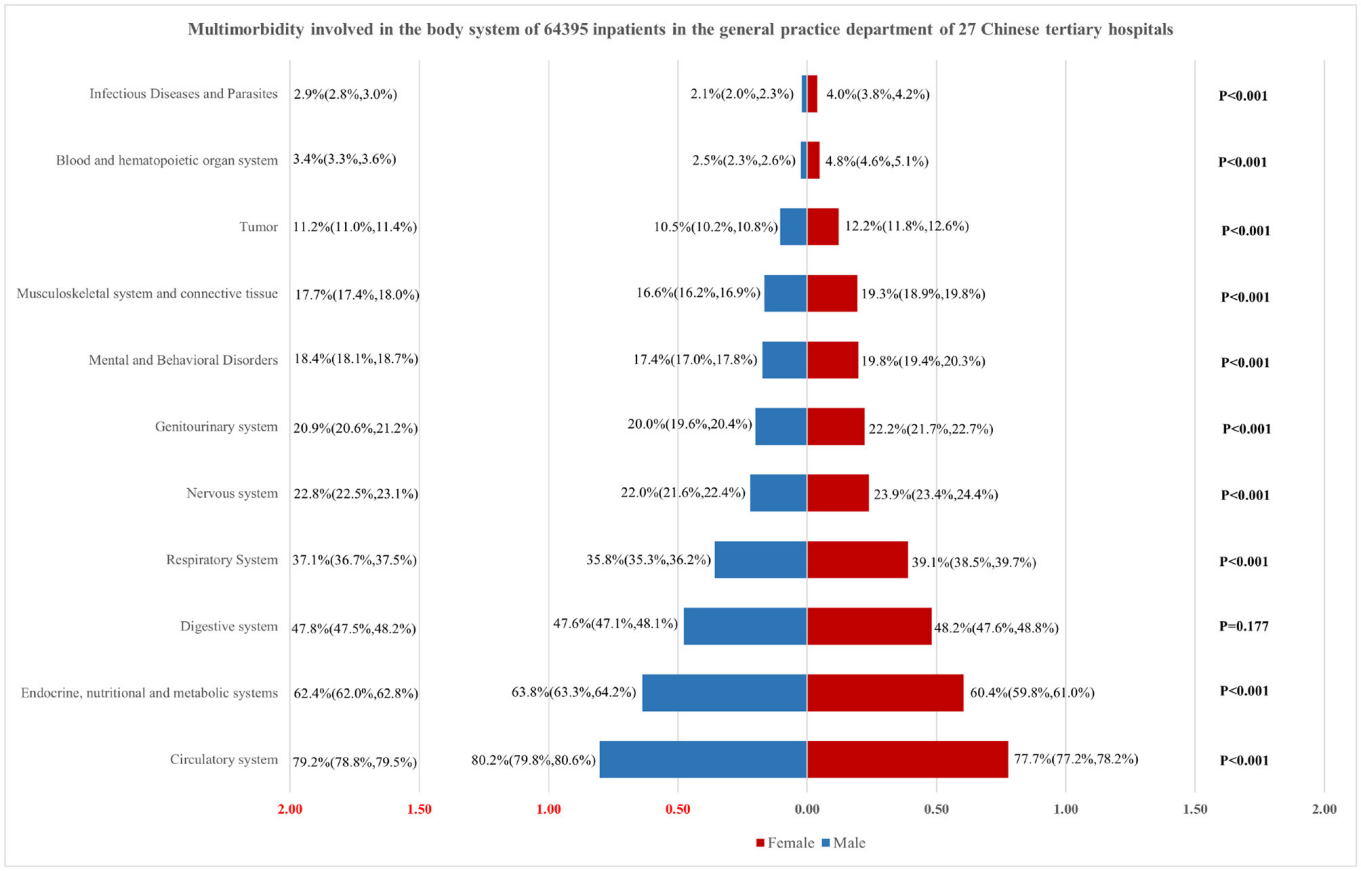
Clinical characteristics of multimorbidity involved in the body system.

The circulatory system was the most frequently associated body system with multimorbidity among 64, 395 hospitalized patients, with a prevalence rate of 79% (95% CI = 78.8–79.5%), which was significantly higher than the prevalence rate for other systems. The other systems associated in multimorbidity include endocrine, nutritional and metabolic systems (62.4%, 95% CI = 62.0–62.8%), digestive system (47.8%, 95%CI = 47.5–48.2%), respiratory system (37.1%, 95%CI = 36.7–37.5%), nervous system (22.8%, 95%CI = 22.5–23.1%), genitourinary system (20.9%, 95%CI = 20.6–21.2%), mental and behavioral disorders (18.4%, 95%CI = 18.1–18.7%), musculoskeletal system and connective tissue (17.7%, 95%CI = 17.4–18.0%) and tumors (11.2%, 95%CI = 11.0–11.4%). In addition, we further examined the association between gender and the involved human body system in multimorbidity. The results showed that female inpatients' health outcomes were worse in Respiratory System, Nervous system, Genitourinary system, Mental and Behavioral Disorders, Musculoskeletal system and connective tissue, Tumor, Blood and hematopoietic organ system, and Infectious Diseases and Parasites (*P* < 0.001). Meanwhile, the male inpatients suffered more Circulatory system, and Endocrine, nutritional and metabolic system problems (*P* < 0.001) (see Figure 1).

## Discussion and Policy Implications

The number of patients with multimorbid condition may continue to rise in developing countries as the population ages, life expectancy increases, and lifestyles change (22, 23). In comparison to patients with a single chronic disease, multimorbid patients have complicated causes, high medical costs, and a low quality of life, resulting in a significant burden on the country, society and family (24–27). In view of this, in December 2016, NICE released the United Kingdom “Multimorbidity: Clinical Evaluation and Management,” stating that the clinical guidelines for a single disease are not applicable to patients with multimorbidity. As part of their overall strategy, medical staff should develop effective management plans for multimorbid patients (28). At present, China and many other developing countries have not issued specific clinical treatment guidelines for multimorbidity. The diagnosis and treatment of multimorbidity are still based on specialist diagnosis. The advantages of general medicine in the field of multimorbidity prevention and treatment have not been systematically studied. This article comprehensively analyzed the clinical epidemiological characteristics and influencing factors of multimorbidity in general medicine inpatients in 27 representative tertiary grade-A hospitals in China, and provides accurate evidence for effective prevention and control of multimorbidity.

Firstly, we believe that establishing a GP-PCIC multimorbidity prevention and control model is critical. “The Mortality, Morbidity, and Risk Factors in China and Its Provinces from 1990 to 2017” published by The Lancet showed that the prevalence of hypertension and diabetes was 25.2 and 9.7% respectively. The overall prevalence of chronic diseases is gradually increasing, indicating that the current state of chronic disease management is still severe (29). Due to the combined effects of multiple chronic diseases, patients with multimorbidity face reduced quality of life, heavy psychological burden, prolonged hospital stay, increased number of readmissions, increased emergency visit rate, high incidence of multiple medications and adverse drug events and waste of medical resources (30–33). This study demonstrates that the prevalence of multimorbidity among inpatients in the general practice department of tertiary hospitals in China is extremely high, reaching 93.1%, which is comparable to the results reported by Ma et al. (34), but significantly higher than that of foreign Ge et al. (35), Gupta et al. (36) and Ibarra-Castillo et al. (37). This may be due to the fact that the study participants were recruited from tertiary hospitals with a relatively high overall complexity and likelihood of the cases. It can be seen that the prevention and control of multimorbid condition should be a primary focus of chronic disease prevention and control.

Based on the experience of developed countries, the establishment of a patient-centered integrated service system (Patient Centered Integrated Care, PCIC) provides comprehensive, continuous, and proactive services for patients with multimorbidity, and raises their awareness on disease risk factors. A healthy lifestyle can help patients live longer, improve their quality of life, alleviate their economic burden, and reduce medical expenses. General hospitals in China are of large-scale, with many departments, qualified medical staff, high-tech equipment, and strong first aid capabilities. However, the cost of diagnosis and treatment is relatively high, characterized with longer waiting times. Additionally, primary medical institutions benefit from increased access to services and cost savings. Therefore, there should be a link between general practice department in tertiary hospitals and general practitioners in primary medical institutions to build a general practitioner-based patient centered integrated service system (General Practitioner Based Patient Centered Integrated Care, GP-PCIC). It is hence expected to play an important role in the clinical diagnosis and treatment and comprehensive prevention and control of multimorbid diseases.

Secondly, two-way referral model between tertiary hospitals and primary hospitals is necessary. As general practice departments in tertiary grade-A hospitals support hierarchical diagnosis and treatment and maintain close ties to grass-roots community health service centers, it is critical to establish a link between grass-roots medical institutions and comprehensive clinical specialties. A chronic disease management system should be established to facilitate the effective sharing of electronic medical records between hospitals and community health services (38). The general practice department of a tertiary hospital should be transferred to a ward or to a lower-level hospital or community health service center for treatment depending on the patient's condition and needs. Patients should receive comprehensive, coordinated, and continuous medical care through chronic disease management platforms. Constructing a hierarchical diagnosis and treatment system is an important measure for the allocation of medical resources and the promotion of the equalization of basic medical and health services. This can be achieved through the integration and sharing of medical and health resources in the medical consortium, innovative health management, medical consortium operation management, hierarchical diagnosis and treatment, and medical insurance payment models.

Meanwhile, comprehensive interventions for multimorbidity in elderly and obese patients from both clinical and healthy lifestyle levels should be strengthened. This study found that old age and obesity are independent risk factors for multimorbidity. As age increases, the prevalence of multimorbidity increases significantly. Similar findings were reported in previous studies (39). The increase in age causes the body's metabolic rate to slow down, the body and organs gradually decline in function, and the possibility of chronic diseases in various body systems increases. Obesity is another major risk factor that increases the risk of chronic diseases. Obesity increases the risk of developing heart disease, hypertension, diabetes and other diseases. The “Report on the Status of Nutrition and Chronic Diseases of Chinese Residents (2020)” issued by the National Health and Family Planning Commission indicates that more than 50% of adult residents in China are overweight and obese (40). As a result, treating multimorbid condition is more complicated than simply diagnosing and treating a single disease. Hence, it is necessary to effectively manage multiple risk factors concurrently. According to the clinical characteristics of patients with multimorbidy, there is a need to explore the connection between clinical treatment and healthy lifestyle. From a practical standpoint, a clinical diagnosis and treatment plan for multimorbid patients is established based on general practitioners, with “patient-centered, clinical, and healthy lifestyle integration” to achieve a hospital-community-family trinity health management model (41).

Relevant studies point out that lifestyle plays an irreplaceable role in improving the health outcomes of most chronic diseases and hence significantly reduces medical expenses. Main interventions may include weight control, reasonable diet, adequate exercise, adequate sleep, smoking cessation and alcohol restriction (42–45) (shown in Supplementary Table 2). In the future, general practitioners should explore healthy lifestyle intervention programs suitable for hospitalized patients with multimorbidity as a core component of clinical treatment programs.

In addition, general practitioners' capacity to diagnose and treat diseases in critical systems and their comprehensive prevention and control capabilities should be emphasized. This study found that the most commonly affected systems of multimorbidity are the circulatory system, endocrine, nutrition and metabolism, digestive system, and respiratory system. Therefore, general practitioners in provincial-level tertiary hospitals should be targeted to improve the clinical treatment of common diseases and frequently occurring diseases in the corresponding system. At the same time, given that multimorbid patients often have multiple health risk factors, general practitioners also need to have the ability to identify, mitigate and control the main system-specific health risk factors. Therefore, the ability of general practitioners to effectively prevent and control multimorbidity should have the following capabilities: (1) Ability to rapidly diagnose diseases and disorders of the circulatory, endocrine, nutrition, and metabolism systems, as well as the digestive, respiratory, and other systems; (2) Ability to respond effectively to patients' family problems (46) and (3) Ability to detect, control, and propose healthy lifestyle intervention programs suitable for the residents.

As a result, the education and training system for general practitioners in China should be updated to better meet the needs of residents regarding the practitioners' diagnostic and treatment capabilities. To be more specific, there is a need to scale up the residency training model (5 + 3) and rapidly increase the number of general practitioners familiar with general medicine concepts, all of which can serve as a foundation for establishing a GP-PCIC multimorbidity prevention and control model. Secondly, on the basis of harmonizing national audit standards and training quality monitoring systems for general practitioner transfer training (47, 48), specific capacity improvement training for specific chronic disease epidemic trends in specific regions should be provided to address the problem of general practitioners in the circulatory system. Thirdly, there is a need for additional training of general practitioners, with an emphasis on establishing and improving mechanisms for comprehensive disease prevention and control.

### Strengths and Weaknesses of the Study

In most health service systems, GPs are classified as primary health care providers. However, in the special health care context of China, GPs in tertiary hospitals play a dual role of service provider and primary general practitioner trainer. Exploring prevalence, risk factors, and approach to control of multimorbidity among hospitalized patients of these GPs working at tertiary hospitals contributes to the improvement of general practice service system in China as a whole. This is the first large scale study to examine the clinical epidemiology of multimorbidity across a broad range of chronic conditions and to investigate the factors that contribute to it over a four-year period in 27 tertiary grade-A hospitals in China. The true status of multimorbid admissions is expected to serve as a decision-making basis for the development of general practice healthcare system and the precise enhancement of general practitioners' diagnostic and treatment capabilities. The study's limitation is that it examined only 27 tertiary grade-A hospitals with the earliest standardized training bases for general practice residents. Hence, subsequent surveys involving multiple centers and a larger sample size should form objectives in future studies. In addition, there are several other factors, such as primary care service utilization and household income (49), which may also play a role in the development of multimorbidity but were not captured in the current study.

## Conclusions

Multimorbidity is likely common among the inpatients attending general practice department of the tertiary grade-A hospitals in China, with significant gender inequity in the involved human body systems. As far as the findings of this article are concerned, the prevention and control of multimorbidity should focus on the elderly and obese people. Effective countermeasures include establishing a GP-PCIC multimorbidity prevention and control model and enhancing the multimorbidity of elderly and obese patients at both the clinical and healthy lifestyle levels. We also call upon timely intervention and improvement in the diagnosis and treatment capabilities and comprehensive prevention and control measures of general practitioners on the circulatory, endocrine, digestive, respiratory and other systems.

## Data Availability Statement

Publicly available datasets were analyzed in this study. The anonymized dataset for this article can be obtained by contacting the corresponding authors via email.

## Author Contributions

ZZ and MS are responsible for the conception and design of the article, data analysis, and thesis writing and revision. ML and JW are responsible for the implementation of the research and data analysis. YM and CS are responsible for the statistical results and analysis and interpretation of the results. JG is responsible for the quality control and the overall review of the article. All authors contributed to the article and approved the submitted version.

## Funding

This study was sponsored by National Natural Science Foundation of China (number 71603132), Key Project of Collaborative Innovation of Zhengzhou (number 20XTZX05015), Joint Project of National Health Commission and Henan Province (number SB201901072), and Annual Cultivation Project of Zhengzhou University (JC21738031). The funders have no role in study design, analysis, and interpretation of the study findings.

## Conflict of Interest

The authors declare that the research was conducted in the absence of any commercial or financial relationships that could be construed as a potential conflict of interest.

## Publisher's Note

All claims expressed in this article are solely those of the authors and do not necessarily represent those of their affiliated organizations, or those of the publisher, the editors and the reviewers. Any product that may be evaluated in this article, or claim that may be made by its manufacturer, is not guaranteed or endorsed by the publisher.
