# Supplementary material for: Multimorbidity in Hospitalized Patients Admitted to General Practice Departments and Its Implications for the General Practice Healthcare System: A Four-Year Longitudinal Study in China
Source: Front Public Health. 2021 Dec 20;9:760792. doi: 10.3389/fpubh.2021.760792 (PMC8720775; doi:10.3389/fpubh.2021.760792)
Supplement: Supplementary file 1 [file Data_Sheet_1.docx]

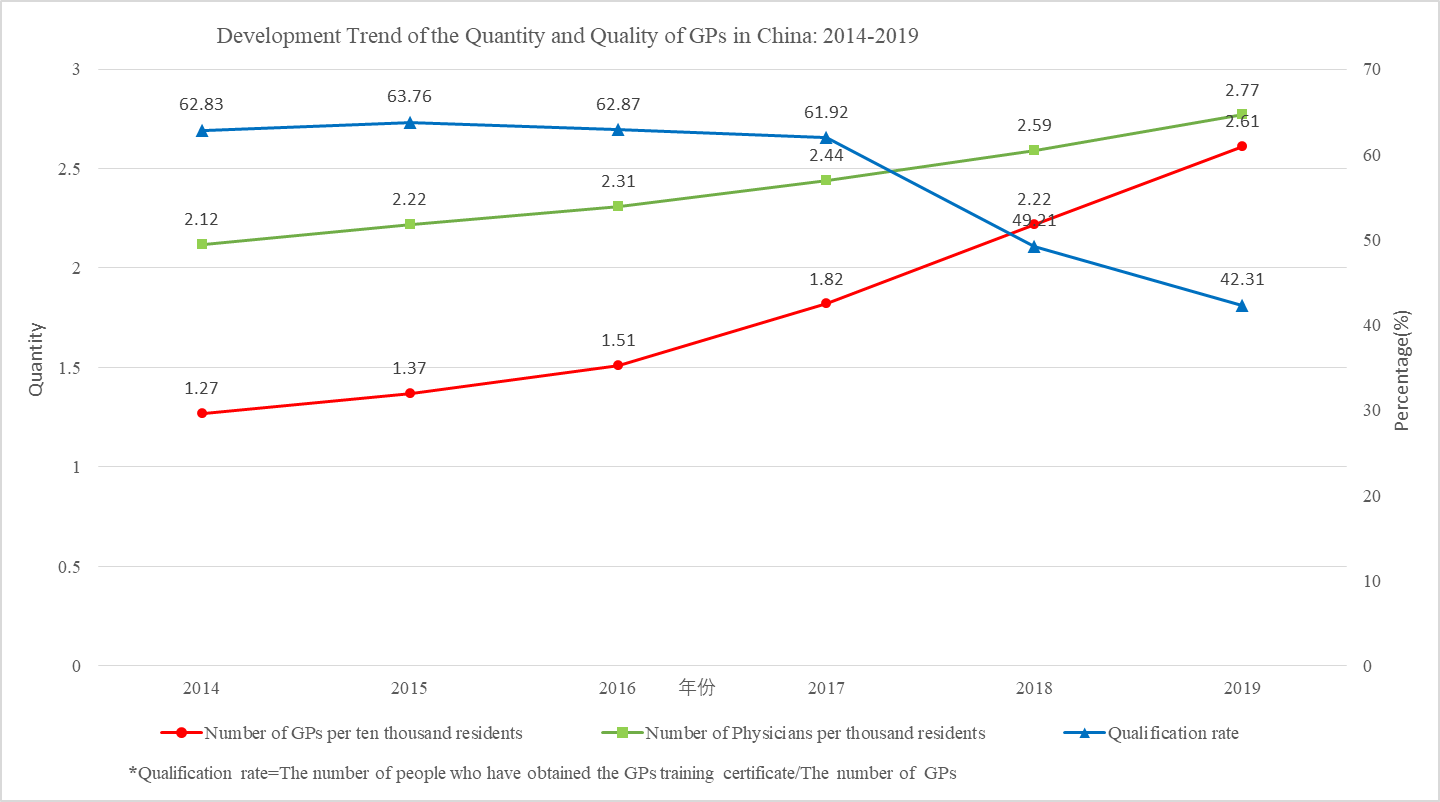


**Figure S1: Development Trend of the Quantity and Quality of GPs in China: 2014-2019**

**Table S1: The 27 tertiary grade-A hospitals that have the earliest standardized training bases for general practice residents in this study**

| Number | Name | Location |
| --- | --- | --- |
| 1 | Peking University People's Hospital | Beijing |
| 2 | Beijing hospital | Beijing |
| 3 | Tianjin People's Hospital | Tianjin |
| 4 | General Hospital of Tianjin Medical University | Tianjin |
| 5 | Hebei Provincial People's Hospital | Shijiazhuang, Hebei Province |
| 6 | The First Affiliated Hospital of Dalian Medical University | Dalian, Liaoning Province |
| 7 | Shengjing Hospital, China Medical University | Shenyang, Liaoning Province |
| 8 | First Affiliated Hospital of Harbin Medical University | Harbin, Heilongjiang Province |
| 9 | Second Affiliated Hospital of Harbin Medical University | Harbin, Heilongjiang Province |
| 10 | Huashan Hospital, Fudan University | Shanghai |
| 11 | Shanghai First People's Hospital | Shanghai |
| 12 | Jiangsu Provincial People's Hospital | Nanjing, Jiangsu Province |
| 13 | First Affiliated Hospital of Zhejiang University | Hangzhou, Zhejiang Province |
| 14 | Anhui Provincial Hospital | Hefei, Anhui Province |
| 15 | Jiangxi Provincial People's Hospital | Nanchang, Jiangxi Province |
| 16 | The First Affiliated Hospital of Nanchang University | Nanchang, Jiangxi Province |
| 17 | Shandong Provincial Hospital | Jinan, Shandong Province |
| 18 | Qilu Hospital, Shandong University | Jinan, Shandong Province |
| 19 | Henan Provincial People's Hospital | Zhengzhou, Henan Province |
| 20 | People's Hospital of Wuhan University | Wuhan, Hubei Province |
| 21 | Hunan Provincial People's Hospital | Changsha, Hunan Province |
| 22 | Xiangya Hospital of Central South University | Changsha, Hunan Province |
| 23 | Guangdong Provincial People's Hospital | Guangzhou, Guangdong Province |
| 24 | The First Affiliated Hospital of Chongqing Medical University | Chongqing |
| 25 | West China Hospital of Sichuan University | Chengdu, Sichuan Province |
| 26 | The First Affiliated Hospital of Lanzhou University | Lanzhou, Gansu Province |
| 27 | Qinghai Provincial People's Hospital | Sining, Qinghai Province |

**Table S2: The 10 dimensions of lifestyle medicine and healthy lifestyle**

| **Project** | **Description** |
| --- | --- |
| **Weight** | Maintain a reasonable weight; |
| **Diet** | Diversified, mainly based on plant-based whole-grain diets; reduce the intake of salt, sugar and sweeteners, oils, red meat, and polished rice noodles; consume supplement vitamins; eat sufficient fruits and vegetables |
| **Exercise** | Regular, appropriate and moderate aerobic exercise; avoid sitting for a long time; do not promote extreme sports (such as marathons) |
| **Sleep** | Sufficient sleep; Ensure that you get at least 7 hours of sleep each night; refrain from staying up late |
| **Smoking cessation** | Do not smoke; avoid secondhand smoke; E-cigarettes should be banned. |
| **Limit alcohol** | The best course of action is to abstain from alcohol consumption; avoid drug addiction and dependence |
| **stress reliever** | Reduce stress levels through yoga, singing, vacations, etc. |
| **Social** | Establish good interpersonal relationships; travel, frequent companion tours and visits for learning |
| **Occupation** | Retirement is delayed, old age has something to do, something to do; learning, continuous learning and education and training; |
| **Physical examination** | Health information collection and evaluation of routine comprehensive items: A baseline assessment should be performed for 35-40 years old; an assessment once in 12-18 months for 40-50 years old (according to the level of cardiovascular risk factors and lifestyle/behavioral status); after 50 years of age, to be evaluated once a year. |
